# From Microbiome Associations to Deployable Precision Probiotics in Animals: A Critical Narrative Review and Proposed Causal Evidence Framework for Next-Generation Probiotic Development

**DOI:** 10.3390/ani16203253

**Published:** 2026-10-16

**Authors:** Shang-Tse Ho, Ming-Ju Chen

**Affiliations:** 1 Department of Wood Based Materials and Design, National Chiayi University, Chiayi City 600355, Taiwan; stho@mail.ncyu.edu.tw; 2 Department of Animal Science and Technology, National Taiwan University, Taipei 10617, Taiwan

**Keywords:** next-generation probiotics, precision probiotics, animal microbiome, multi-omics, culturomics, metabolomics, strain tracking, engineered probiotics, antimicrobial resistance, one health

## Abstract

Next-generation probiotics (NGPs) are promoted as precision tools for animal health, yet the term is often applied to uncommon taxa before causality, safety and target-species efficacy are established. This critical narrative review reframes animal NGPs as a development pathway rather than a taxonomic category. A structured PubMed search was last run on 14 September 2026 with a publication date cutoff of 1 September 2026 and was supplemented by citation tracing and targeted searches of authoritative sources. From the screened literature, 45 representative and conceptually informative sources were purposively selected for the final narrative synthesis; the review was not intended to provide systematic or exhaustive coverage. The detailed synthesis focused on terrestrial farm and companion animals, whereas equine, rabbit and aquaculture studies were treated as boundary evidence. We evaluate how strain-resolved metagenomics, culturomics, metatranscriptomics, metaproteomics, metabolomics, host transcriptomics, machine learning, metabolic modeling and perturbation systems can move discovery beyond association. Studies across animal sectors show many biomarkers but limited causal validation, strain tracking, formulation data, multisite trials and ecological safety assessment. We propose an author-developed conceptual framework comprising a cumulative evidence ladder, from reproducible associations to field-ready deployment, and the ANIMAL-NGP reporting checklist (Animal endotype, Native ecological fit, Identity and genomic safety, Mechanism, Administration and manufacturing, and Longitudinal validation). These tools are intended to align claims with evidence and require prospective application and external validation. Multi-omics should therefore function as an evidence-navigation system, not as a substitute for causality.

## Simple Summary

Probiotics are widely used to support health and performance in farm and companion animals, but the same product does not always work in every animal or production system. One major reason is that animals differ in diet, age, disease stage, antimicrobial exposure, housing, genetics and gut microbiome state. This review explains how next-generation probiotics can be developed more rationally by starting with a defined animal problem, using microbiome and other molecular data to identify a missing or beneficial function, and then testing whether a selected strain or consortium truly causes the expected benefit. We propose a practical checklist and an evidence ladder to help readers judge whether a microbial association is only a biomarker, a promising candidate, or a deployable precision probiotic. The framework emphasizes strain identity, genomic safety, native ecological fit, mechanism, formulation, target-animal trials and long-term safety. Applying these principles can make animal probiotic research more reproducible and may support responsible alternatives to unnecessary antimicrobial use.

## 1. Introduction

Probiotics remain among the most widely used microbiome-directed interventions in animal nutrition and veterinary practice. The internationally accepted concept defines a probiotic as a live microorganism that, when administered in an adequate amount, confers a health benefit on the host [1]. This definition is functional and strain-specific. It does not privilege particular genera or assume that taxonomic novelty alone implies benefit. Nevertheless, commercial animal probiotics have historically relied on organisms that are robust, readily manufactured and familiar in feed or food systems, including lactobacilli, bifidobacteria, *Bacillus* spp., enterococci and yeasts. These organisms can be valuable, but animal responses are often heterogeneous across farms, households, diets, disease stages, ages, breeds and antimicrobial histories.

The term next-generation probiotic (NGP) emerged as sequencing and anaerobic culturing began to identify health-associated commensals outside conventional probiotic collections [2]. In human microbiome research, *Akkermansia*, *Faecalibacterium*, *Bacteroides*, *Roseburia* and other host-associated anaerobes became emblematic NGP candidates. A recent *Nature Reviews Microbiology* synthesis describes this movement toward natural and engineered precision probiotics, with emphasis on improved colonization, safety and clinical efficacy [3]. For animal health, however, the useful definition must be broader and more conservative. A familiar species, such as *Bifidobacterium longum*, may be next-generation when it is selected through an endotype-specific, strain-resolved, mechanistic and target-species workflow. Conversely, an unusual anaerobe should not be called an NGP simply because it is difficult to culture or newly named.

Several recent reviews have summarized broad probiotic applications in livestock and companion animals, host-specific probiotics in canine and feline functional foods, and NGPs in poultry [4,5,6]. These terms overlap but are not interchangeable. In this review, NGP is used as a developmental umbrella for live microbial candidates advanced through strain-resolved, function-led and host-contextual evidence. Precision probiotic describes the intended deployment logic: matching a defined strain or consortium to a defined host endotype, function or response phenotype. Host-specific probiotic describes ecological origin or host adaptation and does not, by itself, establish evidence maturity. A live biotherapeutic product is a therapeutic product-development and regulatory concept, used most explicitly in human medicine, and should not be treated as synonymous with NGP. Engineered living microbes are defined by deliberate genetic modification, whereas synthetic microbial consortia are defined by the designed composition and ecological roles of multiple strains.

The opportunity for a new animal health review is therefore not another catalog of candidate taxa. The unresolved problem is translational. How can omics-guided microbial associations be converted into causally supported, safe, manufacturable and reproducible precision products for species with very different gastrointestinal anatomies and production constraints? Accordingly, this review has three objectives: (1) to describe how multi-omics can support discovery and mechanistic evaluation of microbial candidates; (2) to propose a framework for classifying evidence maturity; and (3) to identify the requirements for translating a microbial candidate into a safe and deployable animal product. The central argument is that NGP status should describe a development paradigm and maturity level, not a microbial label.

## 2. Materials and Methods

### 2.1. Review Design and Information Sources

This critical narrative review was designed to evaluate how microbiome and multi-omics evidence can be translated into causally supported, safe and deployable precision probiotics for animals. It was not conducted as a systematic review or meta-analysis. PubMed was the structured bibliographic search tool. The final search was run on 14 September 2026 and was limited to records published from database inception through 1 September 2026. Google Scholar was used for forward-citation checking, and reference lists of highly relevant primary studies and recent reviews were searched manually. Targeted searches of the European Food Safety Authority (EFSA), U.S. Food and Drug Administration (FDA), and EUR-Lex websites were used to identify definitions and regulatory guidance. These supplementary tools were used for citation tracing and guidance retrieval rather than for calculating the quantitative PubMed yield.

The main synthesis focused on terrestrial farm and companion animals. Human studies were eligible only when they established a general concept, technology or safety principle that could be transferred to animal probiotic development. Aquaculture, equine and rabbit studies were treated as boundary evidence because their delivery routes, environmental exposure, hindgut physiology or management conditions require sector-specific interpretation. Because the shared PubMed animal block did not explicitly include horse, rabbit, fish or aquaculture terms, boundary-sector evidence was identified through targeted supplementary searches during citation tracing using species- and sector-specific terms (e.g., equine/horse, rabbit, aquaculture/fish). These targeted searches were not included in the quantitative PubMed yield.

### 2.2. Search Strategy and Retrieval Results

All concept terms were searched in the PubMed Title/Abstract field. The following animal block was appended to each query: (animal* OR veterinary OR livestock OR dog OR dogs OR cat OR cats OR cattle OR calf OR calves OR pig OR pigs OR piglet* OR poultry OR chicken* OR broiler* OR sheep OR lamb* OR goat* OR ruminant*). Each term in the concept and animal blocks was individually tagged [Title/Abstract] in PubMed. In the abbreviated strings below, [animal block] denotes verbatim substitution of this shared block. Each query also included the publication date filter 1900/01/01:2026/09/01 [Date-Publication]. The hit counts were recorded when the searches were rerun on 14 September 2026.

**Query 1. NGP terminology (40 hits):** (“next-generation probiotic” OR “precision probiotic” OR “host-specific probiotic”) AND [animal block].

**Query 2. Functional omics (848 hits):** (probiotic* OR postbiotic*) AND (metagenom* OR metatranscriptom* OR metaproteom* OR metabolom* OR “multi-omics” OR multiomics OR “holo-omics” OR holoomics) AND [animal block].

**Query 3. Engineered microbial interventions (120 hits):** (“engineered probiotic” OR “engineered probiotics” OR CRISPR OR “synthetic biology” OR nanobody OR nanobodies) AND (probiotic* OR “living therapeutic” OR “live biotherapeutic”) AND [animal block].

**Query 4. Safety and regulation (2043 hits):** (probiotic* OR “live biotherapeutic” OR “direct-fed microbial”) AND (safety OR “antimicrobial resistance” OR regulation OR biosafety OR containment) AND [animal block].

The four PubMed queries returned 3051 records before deduplication. Duplicate records across query sets were removed by PMID, yielding 2802 unique records for title and abstract screening. The broad safety query was retained because genomic safety, antimicrobial resistance, manufacturing and ecological containment are central to the proposed development framework.

### 2.3. Eligibility Criteria and Selection Process

Records were retained when they met at least one of the following criteria: (1) they defined probiotics, postbiotics, next-generation probiotics or live biotherapeutic products; (2) they linked an animal microbiome feature to a defined endotype or candidate function; (3) they reported strain-resolved identity, cultivation, genomic safety, functional omics, mechanistic perturbation, formulation, target-species efficacy or longitudinal safety; or (4) they provided authoritative regulatory or safety guidance applicable to microorganisms used in animals or the food chain. Peer-reviewed original research and reviews in English were eligible. Mechanistic studies outside the principal animal groups were retained only when they supplied a transferable method or causal principle.

Duplicate publications, conference abstracts, non-peer-reviewed commercial material, studies without accessible methodological detail, and papers unrelated to microbial intervention development were excluded. Association-only animal microbiome studies were eligible as discovery evidence but were not interpreted as proof of probiotic efficacy. Titles and abstracts of the 2802 unique PubMed records were screened first, after which potentially informative reports were assessed in full text. Because this work was designed as a critical narrative review rather than a systematic review, full-text inclusion/exclusion counts and coded reasons for exclusion were not prospectively retained. Accordingly, Figure S1 presents a simplified transparency flow based only on documented counts rather than a complete PRISMA-style selection diagram.

The purpose of the review was to develop and illustrate a causal evidence framework rather than to estimate a pooled intervention effect or provide exhaustive coverage of all potentially eligible studies. Source selection therefore emphasized publications informative for key translational domains of animal NGP development, including endotype discovery, strain identity and safety, functional validation, in vivo pathway engagement, causal perturbation, target-species efficacy, formulation/manufacturing, and deployment readiness. The final 45 sources were thus purposively selected as representative examples spanning conceptual definitions, enabling technologies, companion- and farm-animal applications, formulation and safety, and regulatory guidance. Backward and forward citation tracing and targeted searches of authoritative sources were used to address conceptual and regulatory gaps. Study characteristics and the evidentiary role of each source are summarized in Table S1.

Six illustrative sources were associated with the authors’ research group or collaborative network. Authorship was not a selection criterion; these studies were retained because they met the same relevance criteria as other included sources and illustrated distinct aspects of the translational pathway. The potential for selection bias arising from the prominence of author-associated studies is addressed explicitly in Section 10.

To strengthen the reliability of the revised synthesis, both authors independently re-evaluated all 45 retained sources for relevance to the review objectives and appropriateness of their assigned role in Table S1. For candidate-development studies, the authors also independently assessed the highest evidence milestone directly supported by the reported data. Differences in interpretation were resolved by discussion and consensus. This retrospective verification was limited to the final 45-source narrative set and did not constitute duplicate screening of the original 2802 records.

### 2.4. Qualitative Synthesis and Statistical Approach

Because the included literature was highly heterogeneous with respect to animal species, physiological or disease context, microbial interventions, experimental designs, analytical methods, mechanistic questions, and measured outcomes, no meta-analysis, pooled effect-size estimation, or other quantitative synthesis was performed. Evidence was therefore synthesized qualitatively. Descriptive counts, where reported, refer only to the documented PubMed retrieval and to the final 45-source narrative set; they were not used to estimate pooled effects or the relative strength of evidence across studies.

Because the included sources ranged from definitions and microbiome-association studies to mechanistic experiments, target-animal trials, formulation studies, and regulatory guidance, a single formal risk-of-bias instrument was not considered appropriate across all source types. Candidate-development studies were instead appraised using the author-proposed causal evidence ladder according to the following milestones: reproducible association with a defined animal endotype, strain identity and safety, biologically relevant function, in vivo pathway engagement, causal perturbation, controlled target-species efficacy and safety, and field-level deployability.

The initial literature search and title/abstract screening of the 2802 unique PubMed records were conducted by M.-J.C. As described in Section 2.3, during revision both authors independently reassessed the final 45-source narrative set for relevance to the review objectives, appropriateness of the source role assigned in Table S1, and, for candidate-development studies, the highest evidence milestone directly supported by the reported data. Differences in interpretation were resolved through discussion and consensus. Because this verification was retrospective and restricted to the final narrative set rather than designed prospectively as a duplicate-screening or inter-rater reliability study, no inter-rater reliability coefficient was calculated.

Evidence-milestone assignments were treated as qualitative descriptors rather than numerical scores or a validated grading system. The proposed ladder is cumulative at the candidate-development-program level, whereas an individual publication may document only one or several milestones. Accordingly, the study-level evidence mapping reports the highest evidence milestone directly illustrated by each publication and separately identifies limiting or missing prerequisites. A publication demonstrating an advanced milestone does not, by itself, establish that all preceding requirements have been fulfilled. Full cumulative maturity at a given level therefore requires the relevant preceding milestones to be supported across the complete evidence package for the candidate.

Because the proposed evidence ladder is intended primarily to describe the maturity of live microbial candidates, standalone postbiotic interventions were retained as complementary evidence but were not assigned ladder levels. Matched live-versus-postbiotic comparisons could inform interpretation of viability-dependent effects on a live candidate, but such comparisons alone were not considered sufficient to establish pathway-specific causal perturbation.

### 2.5. Operational Definitions

Terminology is essential because probiotic, NGP, live biotherapeutic product, engineered living microbe and postbiotic are not interchangeable. Postbiotics are preparations of inanimate microorganisms and/or their components that confer a health benefit on the host; purified metabolites alone are not postbiotics under the International Scientific Association of Probiotics and Prebiotics (ISAPP) consensus definition [7]. Holo-omics refers to integrated host–microbiota multi-omics and is especially relevant for animal production because the desired endpoint is a host phenotype, not a microbiome profile by itself [8]. Public resources, such as the HoloFood Data Portal, demonstrate how animal-level metadata and linked multi-omics can be organized for cross-study use [9].

For this review, an animal NGP is operationally defined as a live microorganism or rational live microbial consortium developed through strain-resolved, function-led and host-contextual evidence to address a defined animal phenotype or physiological objective. A precision probiotic is an NGP or other probiotic deployed with explicit matching to an endotype, function or response predictor; thus, precision describes the targeting strategy, whereas NGP describes the development pathway used here. Host-specific origin can strengthen ecological fit but is not sufficient to establish NGP status. Live biotherapeutic product terminology is reserved for contexts in which a therapeutic product category is specifically applicable and is not used as a synonym for animal NGP. Engineered living microbes are discussed as an adjacent precision-biotherapeutic class because genetic engineering can add target-specific functions, but engineering alone does not establish probiotic benefit or safety. Postbiotics are treated as complementary products with their own evidence requirements rather than as automatically equivalent non-live versions of probiotics (Table 1).

## 3. A Causal Evidence Concept: From Biomarker to Deployable Product

The recurring weakness in animal microbiome intervention papers is the rhetorical jump from “depleted in disease” to “candidate probiotic.” A microbe may be depleted because inflammation removed its niche, because medication suppressed it, because the host stopped supplying a substrate, or because another trophic partner disappeared. Reintroducing that organism alone may therefore fail. The central translational task is to separate biomarkers of health from drivers of health and to define the conditions under which a driver can be manufactured, delivered and sustained.

**Figure 1:**
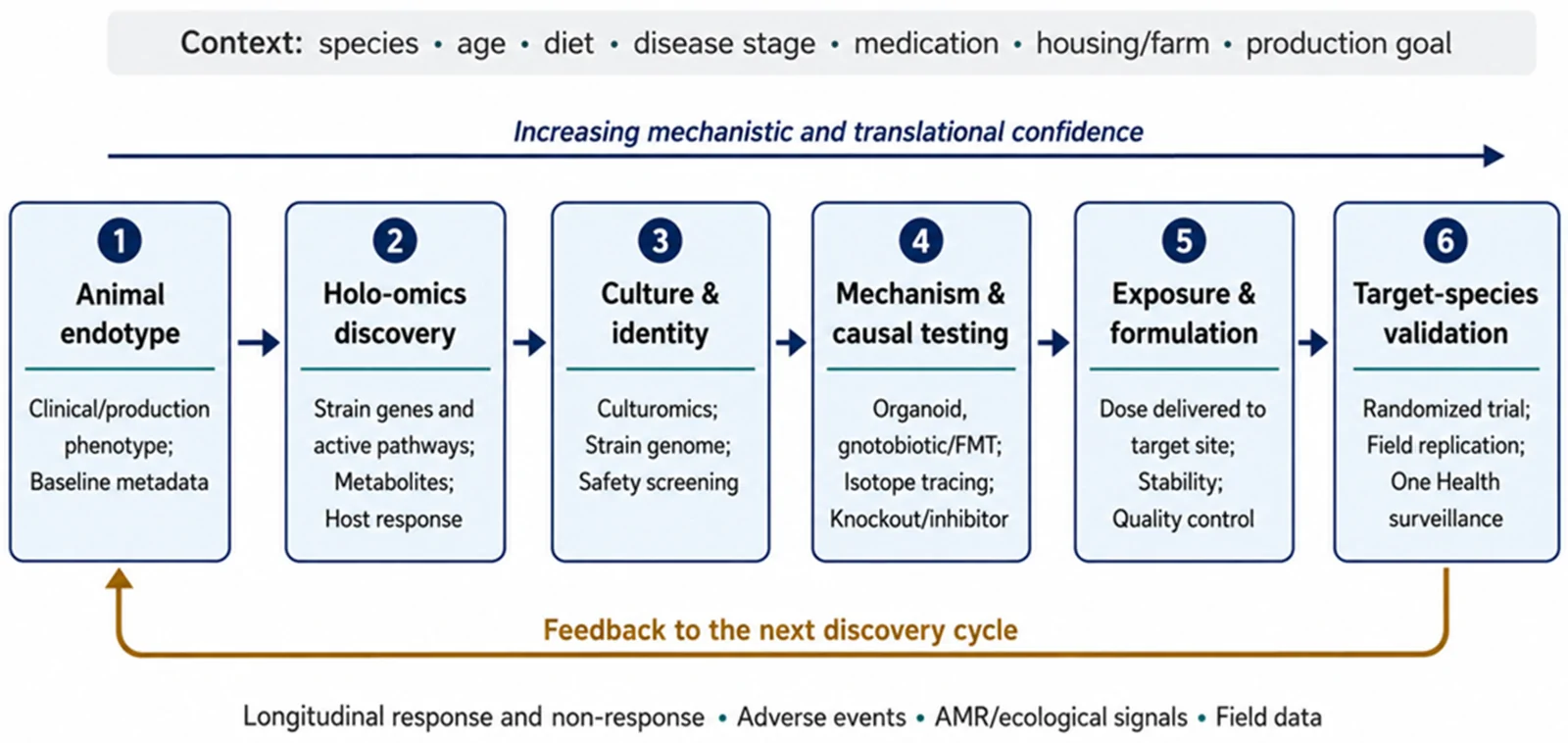
Omics-to-NGP discovery and validation loop. The numbered elements indicate a useful translational logic, not mandatory linear stages; activities can be revisited iteratively as new mechanistic, formulation, safety or field data emerge. Multi-omics adds translational value when it narrows mechanistic hypotheses and is connected to culturing, perturbation, formulation, target-species validation and longitudinal feedback.

We propose that animal NGP development be judged by two coupled axes: causal confidence and deployability. Causal confidence increases when an association is linked to strain identity, activity, metabolite flux, host response and perturbation. Deployability increases when the candidate survives manufacturing and storage, reaches the target site, has acceptable genomic and ecological safety, performs in the target species and is reproducible under realistic farm or household conditions. Figure 1 summarizes this concept as a discovery and validation loop rather than a linear pipeline.

## 4. Multi-Omics as an Evidence-Navigation System

### 4.1. Begin with the Animal Endotype

The most important decision in an NGP program occurs before sequencing. Animals grouped under the same clinical label may have distinct microbial and metabolic states. Disease stage, diet, age, body condition, breed, housing, lactation stage and antimicrobial exposure can all restructure the gut microbiome. A candidate discovered from an unstratified case-control contrast may therefore reflect a confounder or a subgroup-specific response rather than a general therapeutic target.

Canine chronic enteropathy illustrates the value of functional endotyping. In a shotgun metagenomic and targeted metabolomic study of 78 healthy dogs and 138 dogs with chronic enteropathy, dogs with an increased dysbiosis index showed much larger shifts in microbial functional genes and fecal metabolites than dogs with chronic enteropathy but a normal dysbiosis index [10]. A precision probiotic strategy should therefore treat the dysbiosis-index-associated bile acid and carbohydrate-metabolism state as an endotype, not assume that all chronic-enteropathy dogs share the same microbial deficit. This principle also applies to farm animals, where developmental stage, diet transition and management practices can determine both colonization opportunity and response.

### 4.2. Strain-Resolved Metagenomics, Long Reads and Culturomics

16S rRNA gene sequencing remains useful for community screening but is usually insufficient for NGP development because probiotic benefit and safety are strain-specific. Shotgun metagenomics can resolve genes, strain variation, mobile genetic elements, virulence factors and biosynthetic capacity. Long-read or hybrid assemblies are particularly valuable when antimicrobial-resistance genes or virulence determinants must be linked to plasmids, chromosomes or candidate genomes. Large reference resources also expand the discovery space. The 2026 Comprehensive Ruminant Microbial Catalog reconstructed 39,696 strain-level metagenome-assembled genomes from 2325 samples across eight ruminant hosts and mapped vitamin-synthesis potential across major pathways [11]. Such catalogs do not prove probiotic efficacy, but they provide discovery infrastructure for rational cultivation and synthetic-community design.

Metagenomic association becomes actionable only when the organism can be cultured, tracked or manipulated. Targeted culturomics closes this gap by using predicted physiology to design media, anaerobic conditions and co-culture strategies. In preruminant Holstein calves, microbiota and immune-marker correlations were used to prioritize *Bifidobacterium longum* subsp. *longum*, followed by isolation and in vitro screening of host-derived strains [12]. The key lesson is that next-generation status can arise from the discovery and validation workflow, not merely from an exotic genus.

### 4.3. Activity Layers: Metatranscriptomics and Metaproteomics

DNA describes functional potential, not current activity. Metatranscriptomics identifies pathways activated under a diet, infection, disease stage or intervention, whereas metaproteomics can verify production of enzymes, adhesins, transporters, bacteriocins or other effectors. These activity layers are especially important when community composition changes little but function changes substantially. Metaproteomics and artificial-intelligence-assisted protein interpretation have been proposed as underused tools for identifying next-generation probiotic mechanisms, although database incompleteness and host/diet matrix effects remain major limitations [13].

For animal NGP development, activity layers should be hypothesis-driven. If the proposed function is bile acid transformation, carbohydrate degradation, mucus adhesion, antimicrobial secretion or epithelial signaling, the relevant genes, transcripts, proteins and products should be measured in the intended niche. Activity evidence does not prove causality by itself, but it can prevent overinterpretation of DNA-level potential.

### 4.4. Metabolomics, Flux and Host Response

Metabolomics is often a direct route from microbiome observations to host physiology because microbial metabolites can affect epithelial, immune, hepatic, renal and neural pathways. Short-chain fatty acids, secondary bile acids, indoles, aromatic amino-acid derivatives, lactate, polyamines and microbial lipids are recurring mediators across species. However, a metabolite measured in feces is not automatically produced by the taxon with which it correlates. Fecal concentration reflects the balance among production, absorption, host secretion and transit. Stronger source attribution therefore requires targeted pathway genes, culture validation, absolute quantification, stable-isotope tracing, flux modeling or a perturbation experiment.

In dogs, *Peptacetobacter hiranonis* and the bile acid-inducible *baiCD* gene are strongly linked to secondary bile acid formation [14]. In feline chronic kidney disease, a pilot intervention integrated 16S full-length microbiome profiling with host metabolomics and reported responder-associated changes in gut-derived uremic toxins, short-chain fatty acids (SCFAs) and relative abundance of administered *Lactobacillus* mix strains [15]. The authors appropriately characterized these as hypothesis-generating findings, illustrating how metabolomics can identify a mediation chain without overclaiming definitive clinical efficacy.

### 4.5. Holo-Omics, Machine Learning and Metabolic Modeling

Holo-omics integrates host and microbiome molecular layers so that microbial activity can be interpreted in relation to tissue function and animal phenotype [8]. Machine learning can identify multidimensional endotypes and response predictors, but it can also amplify overfitting and data leakage. Repeated samples from the same animal should remain grouped during train/test splitting, and predictive models should be tested across farms, breeds, seasons and diets. Genome-scale metabolic models and community models can rank candidate strains or consortia by predicted substrate use, cross-feeding, metabolite production and competition. Recent modeling work outside veterinary medicine demonstrates how functional rationale can be used to nominate probiotic candidates [16]; animal applications should use the same principle but validate predictions under target diets and host conditions (Table 2).

## 5. Evidence Ladder and ANIMAL-NGP Checklist

To align claims with evidence, we propose a seven-step evidence ladder (Table 3). The ladder is cumulative as a developmental maturity construct. Assignment of a candidate program to a higher level presumes that the relevant preceding milestones are adequately documented, although individual experimental activities need not occur in a rigid chronological order and may be distributed across multiple publications. Level 0 is reproducible association with a defined animal endotype. Level 1 adds isolation or a defined consortium plus strain-resolved identity and safety. Level 2 demonstrates function in vitro or ex vivo under biologically relevant conditions. Level 3 verifies pathway engagement in vivo using functional omics or targeted biomarkers. Level 4 adds causal perturbation. The perturbation methods listed here are not equivalent: gene knockout or a mechanistically matched inhibitor can test pathway necessity more directly; stable-isotope tracing strengthens source and flux attribution; organoids or ex vivo tissues test tissue-level mechanisms; and gnotobiotic or fecal-microbiota-transfer designs test community-level transferability. In cattle and other large livestock, gnotobiotic designs may be impractical or prohibitively expensive. Rumen simulation systems, ex vivo tissue explants, targeted inhibitor or isotope studies, and cross-species transfer models can therefore provide tractable intermediate causal evidence, while remaining explicitly distinct from direct target-species efficacy. Level 5 demonstrates efficacy and safety in a controlled target-species trial. Level 6 adds multisite or field replication, dose/formulation robustness, manufacturing stability, regulatory readiness and post-market ecological surveillance. A product does not need every available technology, but the claimed mechanism and maturity level should match the evidence actually obtained. Because individual papers rarely document the entire cumulative sequence, each representative study is mapped to the highest milestone it directly illustrates; an individual paper is not treated as a complete Level-n certification.

**Figure 2:**
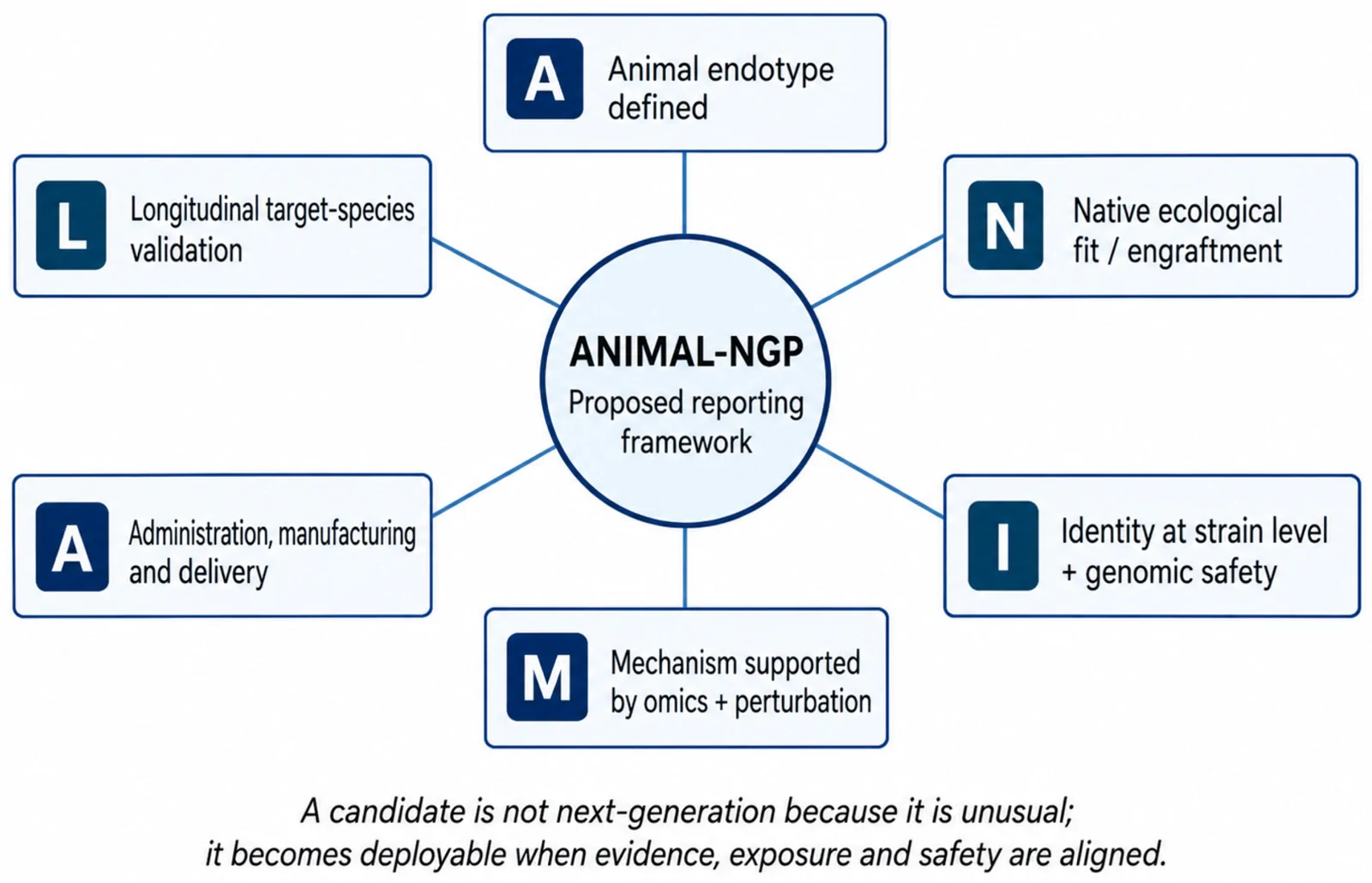
The ANIMAL-NGP checklist. The framework emphasizes completeness of the development pathway rather than taxonomic novelty. The repeated letter A is intentional: the acronym includes both animal endotype and administration/manufacturing as distinct dimensions.

The ANIMAL-NGP checklist translates the ladder into a proposed study design and reporting framework. It is not a regulatory standard or a validated minimum requirement. The checklist emphasizes animal endotype, native ecological fit, identity and genomic safety, mechanism, administration/manufacturing and longitudinal validation (Figure 2).

## 6. Companion Animals: Precision Requires Endotypes and Clinically Meaningful Outcomes

### 6.1. Canine Chronic Enteropathy and Bile Acid Function

Companion animals are a relevant test bed for precision probiotics because they share households and chronic disease patterns with humans but retain species-specific diets, bile acid metabolism and microbial communities. Canine chronic enteropathy shows why an NGP strategy should focus on functional deficits rather than a generic objective such as “increase diversity.” The dysbiosis index integrates quantitative changes in key bacterial groups and can help identify a functional subgroup. Dogs with chronic enteropathy and increased dysbiosis index show marked depletion of secondary bile acid formation and broader metabolic disturbance [10]. *Peptacetobacter hiranonis* and *baiCD* provide a functionally anchored marker for this guild [14].

A strong precision trial would stratify dogs by dysbiosis index and secondary bile acid deficit, administer a defined strain or consortium, verify colonization or functional recovery, quantify bile acid genes and metabolites, and link these changes to clinical remission. Depletion of *P. hiranonis* alone should not trigger single-strain replacement without considering upstream deconjugation, substrate availability, intestinal transit and trophic partners.

### 6.2. Feline Chronic Kidney Disease and the Gut–Kidney Axis

Feline chronic kidney disease is attractive for precision probiotic development because microbial fermentation products can contribute to circulating uremic solutes, whereas saccharolytic fermentation and short-chain fatty acids may support barrier and immune homeostasis. In a 2025 pilot study, two probiotic strains were evaluated in cats with chronic kidney disease using microbiome profiling and host metabolomics [15]. The intervention was associated with gut microbial changes, modest numerical reductions in several gut-derived uremic toxins and responder-associated increases in short-chain fatty acids. Baseline and post-intervention strain abundance appeared related to metabolite response, suggesting colonization efficiency as a possible determinant of precision benefit. Future trials should use prespecified renal endpoints, dietary control, targeted uremic-toxin quantification, absolute microbial measures and kidney-stage stratification.

### 6.3. Canine Atopic Dermatitis and Postbiotic Evidence

Canine atopic dermatitis illustrates both biological promise and evidentiary caution. Recent probiotic studies report changes in gut or skin microbiota, immune markers and clinical scores [17,18]. However, a 2025 systematic review and meta-analysis found no statistically significant pooled effect on the Canine Atopic Dermatitis Extent and Severity Index, fourth iteration (CADESI-4) or pruritus visual analogue scores despite directional improvement in several individual trials [19]. This tension supports biomarker-enriched trials rather than broad claims. Studies should test whether specific gut microbial, short-chain fatty-acid, bile acid or immune endotypes predict response.

Postbiotics are attractive in pet foods because thermal processing, oxygen exposure, storage and owner handling can reduce live-cell viability. A 2026 dog and cat study of a prebiotic–postbiotic supplement reported improvement in digestive signs and fecal microbiota features [20]. Yet a canine postbiotic systematic review and meta-analysis found heterogeneous, underpowered evidence and no consistent significant pooled effects for several outcomes [21]. The translational advantage of postbiotics is manufacturing robustness, not presumed equivalence to living strains. The relevant question is which biological function requires viability.

### 6.4. Boundary Companion Species: Horses and Rabbits

Equine and rabbit studies were not synthesized in depth because hindgut fermentation, colic risk, cecotrophy and management systems create distinct translational requirements. Nevertheless, they represent important future sectors. A 2026 equine gut microbiome review highlights the centrality of hindgut microbial stability in health and disease [22]. Rabbit microbiome work also underscores antimicrobial resistance (AMR) and dysbiosis concerns in a species vulnerable to gastrointestinal disturbance [23]. For these sectors, NGP development should include species-specific fermentation ecology, delivery route, safety margin and welfare outcomes rather than simply transferring dog, pig or poultry assumptions.

## 7. Farm Animals: Host-Derived Discovery Must Meet Production-Scale Validation

### 7.1. Calves and Dairy Cattle

Farm-animal NGPs must work under stronger ecological and economic constraints than most companion-animal products. They must tolerate feed or oral delivery, remain active across changing developmental microbiomes, avoid antimicrobial resistance (AMR) risks and demonstrate value in endpoints, such as diarrhea, morbidity, feed efficiency, milk production, somatic cell count or immune resilience. Calves and dairy cattle provide clear examples of omics-guided discovery. In preruminant calves, Chuang et al. integrated microbiota, fecal scores and immune markers before isolating *B. longum* subsp. *longum* strains with antagonistic and immunomodulatory activity [12]. This is a strong discovery pipeline, but target-calf efficacy and field reproducibility remain necessary.

In dairy cows, rumen microbiome-metabolome studies identified taxa and metabolites associated with mastitis-related inflammatory states [24]. Follow-up work used in vitro ruminal fermentation and cow-to-mouse fecal microbiota transplantation to test whether *Ruminococcus flavefaciens* and *B. longum* subsp. *longum* could alter candidate metabolites and mitigate adverse phenotypes transferred from mastitis-associated microbiota [25]. This strengthens causal plausibility but does not yet demonstrate that oral administration prevents or treats mastitis in cows. A decisive study would include randomized dairy-cow allocation, strain tracking, rumen and host metabolic endpoints, somatic cell count and mastitis outcomes, and farm-level replication.

### 7.2. Pigs

The abrupt dietary, social and environmental changes of weaning make piglets a high-value model for microbiome-directed intervention. In 2026, early-life *Lactobacillus rhamnosus* GG (LGG) administration was evaluated using microbiome profiling, metabolomics, jejunal transcriptomics and organoid experiments [26]. Although LGG is a classic probiotic, the study illustrates a next-generation evidence strategy because host, microbial and tissue mechanisms were integrated and experimentally tested. Fermented-feed studies similarly show opportunities and boundaries. A 2026 study linked composite probiotic fermented feed to growth, intestinal morphology and microbial metabolic changes in weaned piglets [27]. For NGP translation, however, the active unit must be specified as a strain, consortium, fermentation ecology or postbiotic fraction. Host-derived taxa are also entering the swine pipeline; *Mitsuokella multacida* AGMB01124 underwent genomic characterization, probiotic-property screening and safety evaluation as a swine-derived candidate [28].

### 7.3. Poultry

Poultry research spans nearly the full evidence ladder. Discovery studies can identify uncultured functional specialists. A 2026 hologenomic chicken study reconstructed gut genomes and linked a genome-reduced specialist bacterium to growth-associated pathways [29]. Such organisms are high-priority candidates for culturomics and causal testing, but should not be described as deployed probiotics. At the intervention level, *Akkermansia muciniphila*-containing preparations were evaluated in lipopolysaccharide (LPS)-challenged yellow-feathered broilers with microbiome and jejunal transcriptome data [30]. Another study comparing a lactobacilli-based probiotic with its matched postbiotic under a non-starch polysaccharide challenge found that the live probiotic produced stronger transcriptomic reduction of intestinal inflammatory pathways than the postbiotic [31]. This matched comparison supports a viability-dependent difference in biological response rather than assuming equivalence between the live and postbiotic preparations; however, it does not by itself establish pathway-specific causality.

Engineered *Limosilactobacillus reuteri* strains that deliver nanobodies against *Clostridium perfringens* toxins represent a more advanced precision-biotherapeutic paradigm. In a controlled broiler study, engineered strains improved feed conversion and weight gain under subclinical necrotic enteritis, with in situ nanobody expression and linked microbial metatranscriptomic and host gene-expression changes [32]. This approach targets a defined pathogen effector rather than attempting nonspecific immune stimulation, but it also raises additional requirements for genetic stability, containment, horizontal transfer assessment and environmental monitoring. *L. reuteri*-derived postbiotics have also improved growth and intestinal-health readouts in necrotic-enteritis-challenged broilers, supporting a complementary non-live strategy when the intended function does not require persistence [33].

### 7.4. Small Ruminants and Aquaculture Boundary

Early-life diarrhea and weaning stress in small ruminants provide tractable settings for host-derived NGPs. A sheep-rumen-derived butyrate-producing *Clostridium beijerinckii* R8 reduced diarrhea-related outcomes and modified intestinal microbiota and barrier-associated phenotypes in goat kids [34]. Sheep-derived *Lactobacillus johnsonii* M5 improved antioxidant, immune and intestinal-health outcomes in early-weaned lambs [35]. These studies support host origin discovery, but farm-level reproducibility and mechanism-based dose selection remain limited.

Aquaculture was excluded from detailed evidence-level synthesis because probiotic exposure can occur through feed, water and biofilters, and released microbes directly interact with environmental microbial communities. Recent systematic and scoping reviews show substantial evidence for probiotics, prebiotics and synbiotics in aquatic animals but also emphasize heterogeneity, water-quality effects and the need for standardized long-term protocols [36,37]. For aquaculture NGPs, the ecological unit is not only the host gut but also the tank, pond or marine environment (Table 4).

## 8. Engineering, Synthetic Ecology, Formulation and Safety

### 8.1. Engineering Should Solve a Defined Biological Problem

Synthetic biology expands probiotic design from naturally occurring functions to programmed sensing, secretion, pathogen targeting or metabolic conversion. The highest-value animal applications are likely to be functions that are difficult to achieve through diet alone, such as toxin neutralization, localized antigen delivery, removal of harmful metabolites or interference with antimicrobial resistance gene transfer. The broiler nanobody study provides an appropriate logic: define the pathogen effector, engineer local neutralization, verify expression in the intestinal niche and link the intervention to host and performance outcomes [32]. Engineering a strain to broadly boost immunity without a measurable molecular target would be much less defensible.

The mechanistic advantage of engineering is also the safety challenge. A programmed function has a genetic substrate that can mutate, transfer or impose a fitness cost. Veterinary use adds environmental exposure through feces, litter, bedding, manure, water and soil. Engineered strains should therefore be evaluated for genetic stability without antibiotic-resistance selection, mobile-element context, horizontal transfer, environmental persistence and fail-safe containment before field use.

### 8.2. Synthetic Consortia and Ecological Fit

Single-strain interventions can fail when the desired function depends on community metabolism. Defined synthetic communities offer a route to combine primary carbohydrate degraders, lactate utilizers, butyrate producers, bile acid transformers or pathogen-excluding strains. The advantage is functional redundancy and cross-feeding; the cost is more complex manufacturing, quality control, stability testing and attribution of adverse events. Each member requires strain-level identity, genomic safety and a defined ecological role. Pairwise compatibility and community function should be tested under the intended diet, because a consortium that works on a purified laboratory substrate may fail in a pelleted broiler ration, milk replacer, high-starch pig diet or household pet food matrix.

### 8.3. Formulation Is Part of the Exposure Definition

Many attractive NGP candidates are extremely oxygen-sensitive, creating a gap between biological promise and deployable product. A 2026 review of microcomposite delivery systems concluded that pectin- and gellan-based systems and multi-material blends appear promising, whereas alginate alone shows inconsistent protection; importantly, oxygen exposure was rarely quantified explicitly [38]. For strict anaerobes, viable count at manufacture is not the relevant dose. The biologically meaningful exposure is viable cells reaching the intended site after storage, feed processing, gastric passage, bile exposure and oxygen stress. Formulation, therefore, is not an accessory technology but part of the mechanism and should be linked to efficacy data.

### 8.4. Genomic Safety, AMR and Regulation

Whole-genome sequencing has transformed probiotic safety assessment by enabling strain-resolved evaluation of antimicrobial-resistance genes, virulence factors, toxins, mobile elements and metabolic liabilities. A 2025 study of industrial probiotic strains combined phenotypic and genotypic AMR assessment and showed that whole-genome screening can reveal diverse antibiotic resistance gene (ARG) profiles [39]. This reinforces the need to integrate genotype with phenotype. In farm animals, AMR assessment must also extend beyond the administered strain because microorganisms are released into manure and environmental networks. The critical distinction is between intrinsic, non-transferable resistance and acquired resistance located on mobile elements.

**Figure 3:**
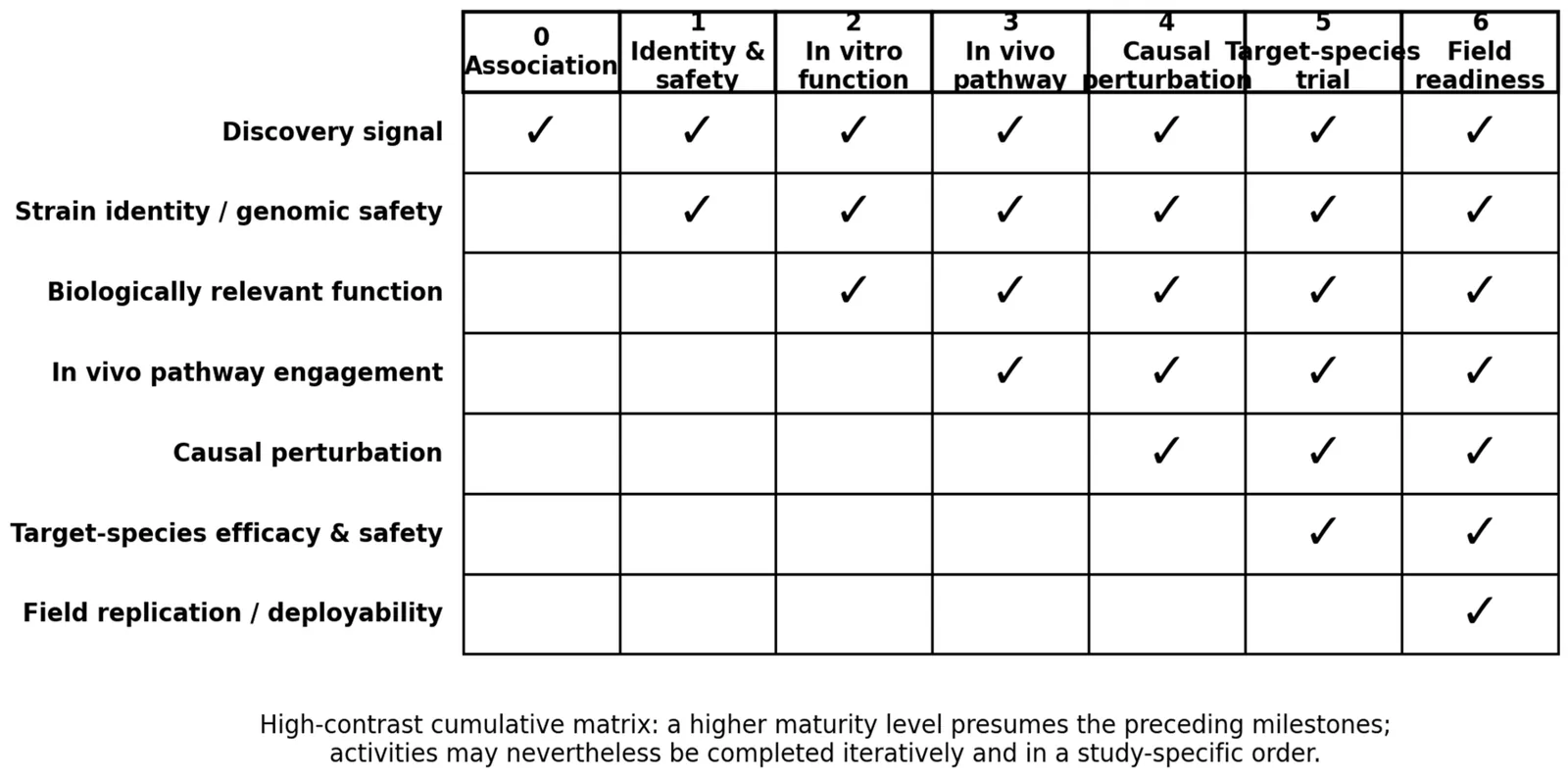
High-contrast cumulative evidence maturity matrix for animal next-generation/precision probiotics. Check marks indicate milestones expected at or below each maturity level. The matrix is intended to align terminology with evidence strength; it does not imply that experiments must be performed in a fixed chronological sequence.

Regulatory language should be precise. EFSA guidance on microorganism characterization for products used in the food chain emphasizes taxonomic identification, whole-genome-based assessment of antimicrobial-resistance determinants, virulence and toxigenicity, characterization of genetic modification, and relevant assessment of viable cells, genetic material and substances of concern in final products [40]. The EFSA Qualified Presumption of Safety (QPS) approach is a pre-assessment route available only when the taxonomic unit is on the QPS list and all applicable qualifications are met; failure to qualify for QPS does not itself mean that a microorganism is ineligible for authorization, but it precludes reliance on the QPS shortcut and requires a fuller case-specific safety assessment [40,41]. In the United States, FDA guidance for live biotherapeutic products concerns human investigational products and should not be automatically applied to veterinary feed microorganisms [42]. Under FDA Center for Veterinary Medicine Compliance Policy Guide Sec. 689.100, direct-fed microbial products bearing disease cure, mitigation, treatment or prevention claims, or non-food structure/function claims, may be considered new animal drugs; products without such claims are regulated according to their ingredient status and intended use [43,44]. In the European Union, microorganisms used as feed additives are evaluated under Regulation (EC) No 1831/2003, including zootechnical functional groups, such as gut flora stabilisers [45]. Figure 3 summarizes the evidence maturity matrix used to align terminology with causal and translational strength. Table 5 presents the corresponding ANIMAL-NGP reporting checklist, whereas Table 6 summarizes frequent reporting gaps and practical remedies for animal NGP manuscripts.

## 9. Research Agenda for the Next Five Years

The animal NGP field is now limited less by sequencing capacity than by experimental architecture. Several priorities could substantially increase the value of future studies. First, trials should be designed around endotypes and longitudinal trajectories. Cross-sectional case-control studies remain useful for discovery but cannot distinguish cause from consequence. Repeated pre-disease and post-intervention sampling is especially important in calves, piglets and broilers, where the microbiome changes rapidly with age. In companion animals, dietary history, medication, disease stage and household environment should be treated as core metadata rather than optional covariates.

Second, absolute quantification and strain tracking should accompany relative-abundance profiles whenever colonization or engraftment is claimed. Relative abundance can rise because another taxon falls. Strain-specific quantitative PCR (qPCR), digital PCR, metagenomic single-nucleotide variants, barcoded experimental strains or culture-based recovery can distinguish exposure, transient passage and persistence.

Third, the mechanism should be tested by perturbation. If the proposed pathway is butyrate production, bile acid conversion, indole signaling, toxin neutralization or immune modulation, the study should include at least one experiment that changes or blocks that pathway. Organoids, ex vivo tissues, defined communities, isotopic substrates, gene knockouts, receptor antagonists or matched inactivated preparations can be more informative than adding a third or fourth descriptive omics layer.

Fourth, multi-omics analysis requires explicit statistical safeguards. The experimental unit should correspond to the unit of treatment allocation and independent replication. This may be the individual animal when animals are individually randomized, but it may instead be the pen, cage, tank or herd when treatment is allocated at group level. Sequencing samples and repeated measurements from the same experimental unit should not be treated as independent replicates. Repeated measures must remain grouped during model training and testing. Batch, cage, pen, farm and household effects should be modeled. Multiple-testing correction should be transparent, and external validation should be expected for predictive models. Reporting only pathways with attractive biological narratives increases false discovery risk.

Fifth, production and formulation variables should be treated as biological variables. Viability at the end of shelf life, after pelleting or mixing, after gastric passage and at the target site can explain biological non-response. Dose should therefore be reported as delivered viable cells or a defined postbiotic composition, not merely as formulation input. For extremely oxygen-sensitive organisms, oxygen exposure during manufacturing and dosing should be quantified.

Sixth, safety needs longitudinal ecological follow-up. The most important risks may not be acute toxicity but persistent colonization, transfer of mobile elements, unexpected metabolic products or dissemination in manure, litter, water, soil and household environments. Longitudinal evaluation should also include animal-welfare outcomes and unintended behavioral or physiological effects when biologically relevant. One Health surveillance is therefore more informative for engineered and host-derived NGPs than a short-term feed-tolerance test alone. Finally, the field needs multisite replication and publication of negative results. A candidate that works only in one diet or microbial background may still be valuable as a precision product, but that boundary should be defined.

## 10. Discussion and Limitations

The proposed framework combines two dimensions that are often considered separately in probiotic research: causal confidence and deployability. It is hierarchical in the sense that the evidence ladder is cumulative, but it is also multidimensional because ecological fit, mechanism, exposure, manufacturing, and safety can mature at different rates. Unlike a formal technology-readiness or regulatory scoring system, the ladder is intended to discipline scientific claims rather than generate a validated numerical score. The ANIMAL-NGP checklist serves a complementary purpose by identifying information that should be reported so that readers can judge where a candidate is strong, incomplete, or context-dependent.

Within the selected literature, evidence was most commonly illustrated at the stages of discovery, association, candidate characterization, and in vivo response, whereas strain/exposure tracking, pathway-specific causal perturbation, multisite target-species efficacy, and long-term ecological safety were less consistently documented. Because the 45 sources were purposively selected rather than systematically sampled, this pattern should be interpreted only as a descriptive feature of the included narrative set and not as an estimate of the distribution of evidence maturity across the broader animal-probiotic literature. Conceptually, the proposed ladder shares with translational-readiness frameworks the principle of staged evidence maturation, but differs by explicitly integrating causal evidence with candidate-specific safety and deployability. It does not replace study-specific causal-inference approaches. Causal-inference methods determine how strongly a particular experimental design supports a causal claim, whereas the proposed ladder describes how that evidence contributes to the overall maturity of a microbial candidate-development program.

A common framework across animal species has practical advantages but also clear limitations. Companion animals, monogastric livestock, ruminants, and aquatic species differ in gastrointestinal anatomy, diet, management, environmental exposure, welfare constraints, and feasible experimental models. In large livestock, some Level 4 perturbation approaches may be impractical; evidence may instead come from rumen simulation systems, ex vivo tissues, targeted isotope-tracing or inhibitor studies, or cross-species transfer models. Such intermediate approaches can strengthen causal inference, but they do not substitute for controlled target-species validation or field replication.

The review process also has limitations. PubMed was the only structured bibliographic database, so relevant animal-science literature indexed mainly in Scopus, Web of Science, or specialized databases may have been missed. The 45 sources were purposively selected for conceptual and translational informativeness rather than as an exhaustive set, and full-text exclusion counts were not prospectively retained. Aquaculture, equine, and rabbit sectors were intentionally treated as boundary evidence rather than synthesized in depth. The original title/abstract screening was performed by one author and was not prospectively duplicated. During revision, both authors independently reassessed the final 45-source narrative set and the evidence-milestone assignments, with differences resolved by consensus; however, this retrospective verification does not substitute for prospective duplicate screening or formal external validation, and no inter-rater reliability coefficient was calculated. The evidence-milestone mapping therefore remains an author-developed interpretive exercise rather than a validated rating instrument. Six illustrative sources [10,12,14,15,24,25] were associated with the authors’ research group or collaborative network. They were retained because they met the same relevance criteria and illustrated different parts of the discovery-to-mechanism pathway, but their prominence in a purposively selected narrative set may introduce selection bias. These examples should therefore be regarded as case studies illustrating the framework, not as independent validation of the framework or of the authors’ own candidate strains.

The framework itself has additional limitations. It has not yet been prospectively applied across independent research groups, and the ANIMAL-NGP checklist has not undergone external validation or reliability testing. The regulatory discussion is weighted toward European Union and United States guidance and should not be interpreted as a globally harmonized regulatory pathway; jurisdiction-specific requirements may differ substantially. Finally, field deployment depends not only on scientific maturity but also on economic feasibility. Strain deposition and banking, specialized manufacturing, stability testing, quality control, regulatory documentation, and acceptable cost-benefit ratios can determine whether a technically mature candidate is adopted. These constraints should be evaluated alongside efficacy, safety, and One Health considerations.

## 11. Conclusions

Next-generation probiotics should not be defined by an exotic taxonomic name, by the use of sequencing or by the presence of a CRISPR cassette. As an evidence-based operational definition proposed in this review, an animal NGP is a strain or rationally designed microbial community selected for a defined animal endotype, characterized at strain level, linked to a mechanistically relevant function and advanced through causal and target-species validation, with manufacturing and safety considered from the beginning.

The current animal literature contains the building blocks of this paradigm. Canine chronic enteropathy demonstrates functional endotyping; feline chronic kidney disease illustrates microbiome-host metabolome integration; calves and dairy cows show microbiome-guided candidate discovery; pig studies link microbial intervention to tissue transcriptomics and organoids; poultry research spans uncultured functional specialists, live-versus-postbiotic comparisons and engineered toxin-targeting strains; and small-ruminant studies demonstrate host-derived candidates for early-life resilience. What remains scarce is the final bridge from mechanistic promise to reproducible, multisite target-animal efficacy.

Multi-omics will be most transformative when it reduces uncertainty rather than increases data volume. The author-proposed evidence ladder and ANIMAL-NGP checklist provide a practical way to align claims with evidence, but they require prospective application, inter-rater testing and independent external validation before they can be considered standardized assessment tools. Deployment also depends on economic feasibility, including costs of strain deposition and banking, manufacturing under controlled conditions, batch quality control, formulation, regulatory dossiers and the value proposition for farmers, veterinarians and animal owners. Addressing these scientific, regulatory and economic dimensions together can move animal probiotics from empirical supplementation toward precision microbiome interventions that are testable, manufacturable, ecologically responsible and compatible with One Health goals.

## Figures and Tables

**Table 1:** Operational distinctions used in this review.

Category	Defining Feature	Development Logic	Evidence Expected to Support the Claim	Main Translational Issue
Conventional probiotic	Live strain with demonstrated benefit in a host context	Empirical or phenotype-based screening	Strain identity, dose and target-host benefit	Context-dependent efficacy and strain specificity
Natural NGP candidate	Live microbial candidate, often host-associated, nominated through strain-resolved microbiome and function-led evidence; not defined by taxonomic novelty	Endotype/function -> isolate -> mechanism -> validate	Strain-resolved identity and safety plus evidence linking the candidate to a relevant function; target-species validation is required for deployment	Cultivation, ecological fit, exposure and reproducibility
Precision probiotic	A probiotic (conventional or NGP) intentionally matched to a defined host endotype, function or response predictor	Define endotype/predictor -> match intervention-> test response -> validate	Strain-specific target-host benefit plus evidence supporting the matching or responder strategy	Biomarker validation, responder heterogeneity and generalizability
Host-specific probiotic	Strain isolated from, or ecologically adapted to, the intended host species or niche	Host-source isolation -> ecological-fit testing -> efficacy	Host origin plus strain identity and demonstrated target-host benefit; host origin alone is insufficient	Ecological origin does not establish mechanism, efficacy or evidence maturity
Synthetic microbial consortium	Defined community with complementary ecological roles	Network and cross-feeding design	Member-level safety, community stability and functional redundancy	Manufacturing complexity and ecological stability
Engineered living microbe	Genetically modified/edited microorganism with programmed activity	Target pathway -> genetic circuit -> containment -> efficacy	Genetic stability, biocontainment, off-target risk and target-host benefit	Regulatory burden and environmental containment
Live biotherapeutic product (LBP)	Live microorganism developed as a therapeutic biological product under a defined regulatory framework; the term is most explicitly established for human investigational products	Therapeutic indication -> product/CMC control -> clinical development -> regulatory review	Jurisdiction-specific quality, safety and efficacy package	Should not be treated as synonymous with animal NGPs or automatically transferred to animal-feed regulation
Postbiotic	Preparation of inanimate microorganisms and/or components with demonstrated benefit; definition follows the ISAPP consensus [7]	Process definition -> bioactivity -> target-host test	Composition/process control and target-host benefit	Mechanistic attribution; may not reproduce live-cell functions

**Table 2:** Contributions and limitations of omics layers in animal NGP development.

Layer	Primary Question	Useful Outputs	High-Value Role	What It Cannot Prove Alone
Strain genomics/metagenomics	What organisms and genes are present?	Strain genomes, metagenome-assembled genomes (MAGs), pathways, antibiotic resistance genes (ARGs), virulence genes, mobile elements	Candidate nomination, safety and tracking	Presence is not activity or benefit
Metatranscriptomics	Which microbial functions are active now?	Pathway expression and in situ stress/effector responses	Confirm activity in the target niche	RNA expression is not causal sufficiency
Metaproteomics	Which microbial and host proteins are produced?	Enzymes, adhesins, effectors and host response proteins	Effector confirmation	Coverage and database bias can mislead
Metabolomics	Which biochemical outputs change?	SCFAs, bile acids, indoles, polyamines and lipids	Bridge microbiota to host physiology	Source attribution and absorption remain uncertain
Host transcriptomics/proteomics	How does the animal respond?	Barrier, immune, metabolic and stress pathways	Connect intervention to tissue phenotype	Tissue timing and specificity matter
Machine learning/metabolic modeling	Can response be predicted or mechanisms prioritized?	Endotypes, response scores and flux/network predictions	Trial stratification and candidate ranking	Classifier performance is not mechanism

**Table 3:** Proposed animal NGP evidence ladder.

Level	Evidence Milestone	What Can Reasonably Be Claimed	What Remains Unresolved
0	Reproducible association with a defined animal endotype	Biomarker or candidate function	Direction of causality
1	Cultured strain or defined consortium plus strain-resolved genome and safety screen	Traceable candidate	Whether function is relevant in vivo
2	In vitro or ex vivo function under biologically relevant conditions	Plausible functional mechanism	Engagement in the intact animal
3	In vivo multi-omics or targeted biomarkers confirm pathway engagement	Mechanistically supported candidate	Causal necessity and sufficiency
4	Hypothesis-matched causal perturbation: e.g., gene knockout/matched inhibitor (necessity), isotope tracing (source/flux), organoid/ex vivo tissue (tissue mechanism), or gnotobiotic/FMT design (community transfer)	Causal support for a defined pathway; strength depends on perturbation design	Target-species therapeutic or production efficacy
5	Controlled target-species trial with prespecified endpoints and strain/exposure tracking	Efficacy and safety under defined conditions	Generalizability and production robustness
6	Multisite/field replication, manufacturing stability, regulatory dossier and ecological follow-up	Deployable precision probiotic or precision biotherapeutic	Post-market ecological and AMR surveillance

**Table 4:** Representative animal studies positioned on the proposed evidence ladder.

Host/Condition	Intervention or Discovery Target	Omics/Mechanistic Design	Cumulative Status/Limiting Prerequisites	Highest Milestone Illustrated *
Dogs/chronic enteropathy	Dysbiosis-index endotypes; *P. hiranonis*/bile-acid guild	Shotgun metagenomics, quantitative PCR (qPCR) dysbiosis index and targeted metabolomics	Level 0 is directly illustrated: a reproducible functional endotype/association is defined, but no cultured candidate or probiotic intervention is tested.	0
Cats/chronic kidney disease	Two probiotic strains/*Lactobacillus* mix	Full-length microbiome profiling and host metabolomics	Illustrates Level-3-type in vivo pathway engagement after intervention. The report does not by itself document the complete lower-level package (e.g., full strain-resolved genomic safety) or Level 5 controlled efficacy/exposure tracking; therefore this is not a cumulative Level 3 certification.	3
Calves/diarrhea	Host-derived *B. longum* subsp. longum isolates	Microbiota, immune markers, culturing and in vitro function	Illustrates Level-2-type isolation and biologically relevant in vitro function. Target-calf in vivo pathway engagement and efficacy remain absent; full cumulative Level 2 also requires the relevant Level 1 identity/safety evidence to be documented across the program.	2
Dairy cows/mastitis-associated state	*R. flavefaciens* + *B. longum* subsp. longum	Rumen microbiome/metabolome, in vitro fermentation and cow-to-mouse fecal microbiota transplantation	Illustrates Level-4-type perturbation/transfer evidence and strengthens causal plausibility. Direct controlled cow intervention is absent, and the paper is not treated as cumulative Level 4 certification unless the preceding identity, safety and functional prerequisites are documented elsewhere in the program.	4
Piglets/weaning stress	*L. rhamnosus* GG	Microbiome, metabolomics, jejunal transcriptomics and organoids	Illustrates Level-4-type tissue-mechanistic perturbation alongside a target-species intervention. Complete strain/exposure tracking, all lower-level safety documentation and field robustness are not established in this report.	4
Broilers/non-starch polysaccharide challenge	Lactobacilli probiotic vs. matched postbiotic	Microbiota, metabolome and multi-tissue transcriptomics	Illustrates Level-3-type in vivo pathway engagement, with the matched live-versus-postbiotic comparison providing evidence of viability-dependent differences in biological response. However, the design does not establish pathway-specific necessity or sufficiency and is therefore not classified as Level 4 causal perturbation. Complete strain identity/safety, exposure tracking, controlled efficacy, and field replication are not all documented in this report.	3
Broilers/subclinical necrotic enteritis	Engineered *L. reuteri* nanobody vectors	Microbial metatranscriptomics, host expression, histology and performance	Illustrates Level-5-type controlled target-species efficacy with in situ programmed activity. Full cumulative Level 5 requires the complete preceding identity, safety, formulation and exposure package across the development program; multisite/ecological Level 6 evidence remains incomplete.	5
Goat kids/diarrhea	*C. beijerinckii* R8	Host-derived isolate, microbiota and barrier/immune phenotypes	Illustrates Level-3-type in vivo pathway/phenotype engagement. Complete lower-level strain-safety documentation, direct causal perturbation and exposure tracking are not all established in this report.	3
Lambs/early weaning	*L. johnsonii* M5	Host-derived strain, antioxidant/immune and intestinal outcomes	Illustrates Level-3-type in vivo pathway/phenotype engagement. Complete lower-level strain-safety documentation, causal perturbation, exposure tracking and field replication are not all established in this report.	3

* “Highest milestone illustrated” indicates the most advanced type of evidence directly demonstrated in the cited study. Because the evidence ladder is cumulative at the candidate-development level, this designation does not imply that a single publication satisfies all preceding milestones. Full maturity at a given level should be determined from the complete evidence package for the candidate, including the relevant earlier requirements for identity, safety, function, mechanism, and validation.

**Table 5:** Proposed ANIMAL-NGP reporting checklist for future studies.

Element	Recommended Information to Report	Why It Matters
A—Animal endotype	Species/breed, age or production stage, disease definition/stage, diet, antimicrobial and medication history, housing/farm, baseline microbiome/metabolites when relevant	Defines the biological target and enables responder analysis
N—Native ecological fit	Source of strain, target niche, substrate dependencies, oxygen tolerance, persistence/engraftment or functional activity in the target site	Prevents selection based only on association
I—Identity + genomic safety	Complete or near-complete genome, strain identifier/deposit, ARG/virulence/toxin/mobile-element analysis and relevant phenotypes	Makes the intervention traceable and safety assessment reproducible
M—Mechanism	Predefined pathway, at least one functional omics or targeted readout, and preferably a perturbation or mediation test	Separates mechanism from correlation
A—Administration/manufacturing	Delivered dose, viability or defined postbiotic composition, carrier, storage, processing stability, release site and batch quality control	Defines true biological exposure
L—Longitudinal validation	Prespecified target-animal endpoints, repeated sampling, withdrawal/persistence when relevant, adverse events, animal-welfare outcomes, unintended behavioral or physiological effects when relevant, non-responder analysis and external replication plan	Establishes durability, safety, welfare impact and generalizability

**Table 6:** Frequent missing elements that should be added to animal NGP manuscripts.

Common Gap	Why It Weakens the Paper	Practical Remedy
Taxonomy-driven claim of “NGP”	Unusual species identity does not demonstrate benefit	State evidence level and avoid calling association-only taxa probiotics
No absolute quantification	Relative abundance may rise because other taxa fall	Add qPCR, digital PCR, spike-ins, culture recovery or strain-specific metagenomic SNVs
No strain tracking	Administration cannot be distinguished from engraftment or passage	Use strain-specific markers, culture plus genome confirmation or experimental barcodes
No perturbation	Multi-omics remains descriptive	Use organoids, ex vivo tissues, isotope tracing, knockout, FMT rescue, inhibitors or matched postbiotics
Formulation not linked to exposure	Viability at manufacture may not equal dose at target site	Measure viability after storage, feed processing, gastric/bile exposure and delivery
Short safety window	Ecological or AMR effects may be delayed	Add longitudinal follow-up of adverse events, animal-welfare/behavioral or physiological effects when relevant, persistence, ARGs and environmental dissemination

## Data Availability

No new data were created or analyzed in this study. Data sharing is not applicable to this article.

## Supplementary Materials

The following supporting information can be downloaded at: https://www.mdpi.com/article/10.3390/ani16203253/s1: Figure S1. Simplified transparency flow for the critical narrative review; Table S1. Characteristics and evidentiary role of the 45 sources included in the critical narrative synthesis (references [1,2,3,4,5,6,7,8,9,10,11,12,13,14,15,16,17,18,19,20,21,22,23,24,25,26,27,28,29,30,31,32,33,34,35,36,37,38,39,40,41,42,43,44,45]).
