# Supplementary material for: From Microbiome Associations to Deployable Precision Probiotics in Animals: A Critical Narrative Review and Proposed Causal Evidence Framework for Next-Generation Probiotic Development
Source: Animals (Basel). 2026 Oct 16;16(20):3253. doi: 10.3390/ani16203253 (PMC13700747; doi:10.3390/ani16203253)
Supplement: Supplementary file 1 [file animals-16-03253-s001.zip › animals-4586725-supplementary.pdf]

Supplementary Materials

From Microbiome Associations to Deployable Precision Probiotics in Animals

Figure S1. Simplified transparency flow for the critical narrative review. The diagram intentionally reports only counts that were prospectively documented and therefore does not imply PRISMA-level systematic-review bookkeeping.

Figure S1. Simplified transparency flow for the critical narrative review

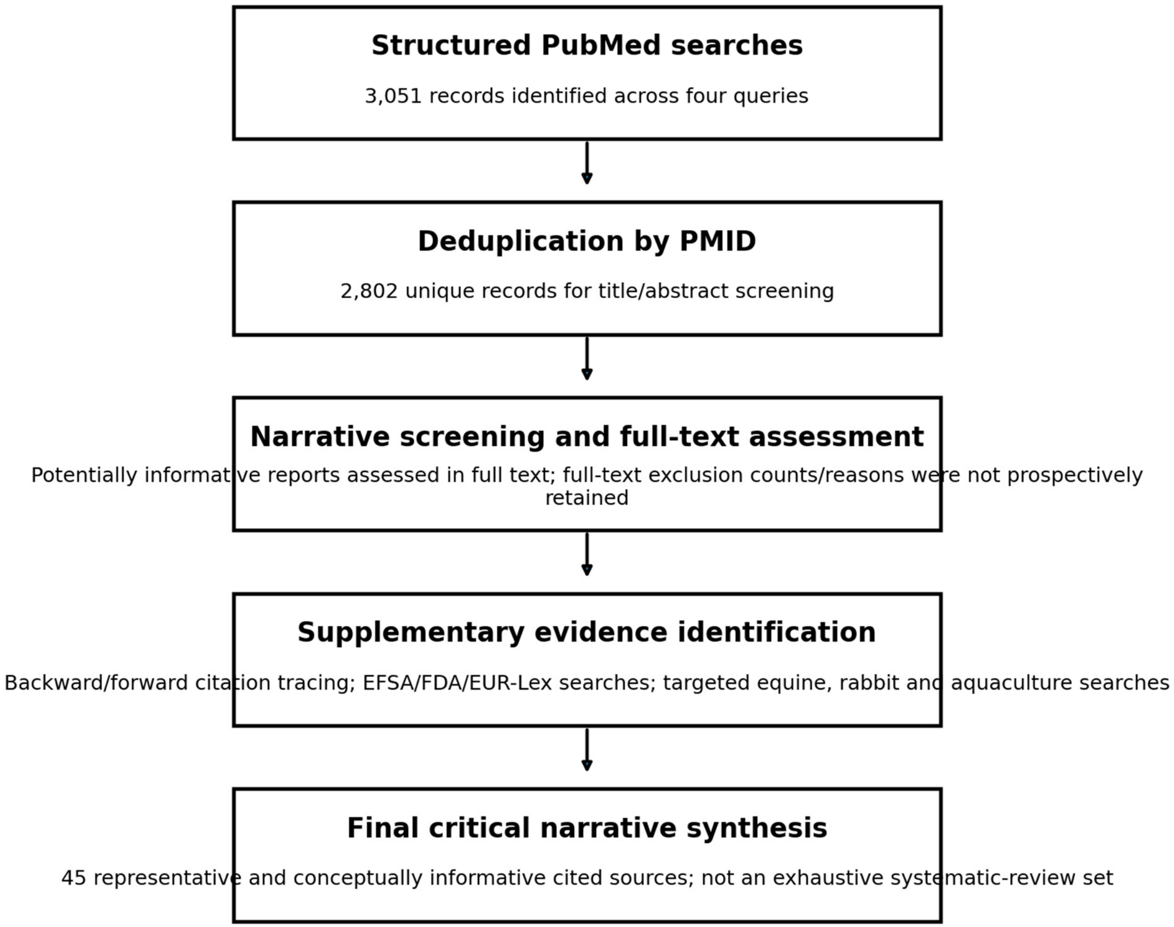

Table S1. Characteristics and evidentiary role of the 45 sources included in the critical narrative synthesis. "N/A" indicates that the source was used for definitions, methods, review-level context, boundary-sector context, formulation/regulation, or complementary non-live/postbiotic evidence rather than to map a live microbial candidate on the proposed evidence ladder. For empirical candidate-development studies, the "Highest evidence milestone illustrated" column identifies the most advanced type of evidence directly demonstrated by that publication; it is not a certification that the paper independently documents every preceding prerequisite in the cumulative ladder.

Ref.	Source category	Species/sector	Study design / source type and intervention/candidate, where applicable	Principal role or outcome in this review	Highest evidence milestone illustrated	Rationale for classification
1	Definition/consensus	General	ISAPP consensus statement	Defines probiotic terminology	N/A	Conceptual definition, not a candidate-development study
2	Conceptual review	Human/general	Narrative perspective	Defines spectrum from probiotics to live biotherapeutics	N/A	Terminology/background source
3	Conceptual review	Human/general	State-of-the-art review	Natural and engineered precision probiotic development	N/A	Translational background source
4	Animal probiotic review	Livestock & companion animals	Narrative review	Broad mechanisms and applications	N/A	Contextual review, not assigned an evidence level
5	Host-specific probiotic review	Dogs & cats	Narrative review	Host-specific/precision nutrition concepts	N/A	Contextual review
6	NGP review	Poultry	Narrative review	Conventional, engineered and synbiotic approaches	N/A	Sector-level context
7	Definition/consensus	General	ISAPP consensus statement	Defines postbiotics and scope	N/A	Conceptual definition
8	Methods/concept	General	Holo-omics framework	Integrates host and microbiota multi-omics	N/A	Methodological concept
9	Data resource	Animal production	Database/resource report	HoloFood linked host-microbiota datasets	N/A	Enabling resource
10	Discovery study	Dogs / chronic enteropathy	Shotgun metagenomics + targeted metabolomics	Defines dysbiosis-index functional endotype	0	Reproducible association/endotype; no intervention
11	Resource study	Ruminants	Large metagenomic catalog	Strain-level genomes and vitamin synthesis potential	N/A	Discovery infrastructure, not an intervention

12	Discovery/isolation study	Preruminant calves / diarrhea	Microbiota + immune markers + culturing + in vitro screening	Host-derived *B. longum* candidate development	2	Cultured traceable strains with biologically relevant in vitro function; target-calf efficacy absent
13	Methods review	General	Metaproteomics/AI perspective	Activity-layer approaches for probiotic mechanism	N/A	Methodological context
14	Association/function study	Dogs	qPCR/bile-acid correlation	Links *P. hiranonis*/baiCD with secondary bile acids	0	Functional association; no intervention
15	Pilot intervention	Cats / CKD	Full-length microbiome + host metabolomics	Responder-associated microbiome/metabolite changes after probiotic mixture	3	Illustrates in vivo pathway engagement after intervention; not a cumulative Level 3 certification because complete lower-level genomic-safety and later controlled-efficacy and exposure-tracking criteria are not all documented in this paper.
16	Modeling study	Human vaginal microbiome	Genome-scale metabolic modeling	Nominates candidate probiotic taxa/functions	N/A	Transferable modeling example outside principal animal scope
17	Intervention study	Dogs / atopic dermatitis	Probiotic intervention + microbiome/clinical outcomes	Reports microbiome and clinical changes	3	Target-animal in vivo response; causal mechanism not established
18	Intervention study	Dogs / atopic dermatitis	Probiotic strains + gut/skin microbiome + clinical/immune outcomes	Adjuvant therapeutic effects and microbiome changes	3	Target-animal pathway/phenotype evidence; limited causal perturbation
19	Systematic review/meta-analysis	Dogs / atopic dermatitis	Meta-analysis	No significant pooled CADESI-4/pruritus effect	N/A	Evidence-synthesis source used to calibrate efficacy claims
20	Intervention study	Dogs & cats / digestive health	Prebiotic-postbiotic supplement	Digestive signs and fecal microbiota changes	N/A	Target-animal effects of a prebiotic-postbiotic supplement; retained as complementary non-live intervention evidence and not mapped to the live-candidate evidence ladder.

21	Systematic review/meta-analysis	Dogs	Meta-analysis of postbiotics	Heterogeneous evidence and inconsistent pooled effects	N/A	Evidence-synthesis source
22	Boundary-sector review	Horses	Review	Equine gut microbiome health/disease	N/A	Boundary evidence only
23	Boundary-sector study	Rabbits	Functional AMR profiling	AMR landscape in rabbit gut microbiome	N/A	Boundary discovery/safety evidence
24	Discovery study	Dairy cows / mastitis-associated state	Rumen microbiome + metabolome	Identifies mastitis-associated taxa/metabolites	0	Association/endotype evidence
25	Mechanistic transfer study	Dairy cow-derived microbiota / mouse model	In vitro rumen fermentation + cow-to-mouse FMT	Tests microbiome-metabolome relationships and candidate strains	4	Illustrates perturbation/transfer evidence supporting causality; direct cow efficacy is absent and cumulative Level 4 depends on complementary documentation of preceding prerequisites.
26	Mechanistic intervention	Piglets / weaning stress	Microbiome + metabolomics + jejunal transcriptomics + organoids	LGG linked to barrier/inflammatory mechanisms	4	Illustrates target-animal intervention plus organoid/tissue mechanistic perturbation; complete lower-level safety/exposure documentation and Level 5 controlled target-species efficacy requirements are not all established; Level 6 field robustness also remains untested
27	Intervention study	Weaned piglets	Composite probiotic fermented feed + microbiome/metabolome	Growth and intestinal health responses	3	In vivo target-animal pathway/phenotype evidence; active unit/causal attribution incomplete
28	Candidate characterization	Swine	Isolation + genome/safety + probiotic-property screening	Mitsuokella multacida host-derived candidate	2	Traceable candidate with safety and in vitro function; target-animal efficacy absent

29	Discovery study	Chickens	Hologenomic/metagenomic analysis	Links specialist bacteria to growth-associated functions	0	Association/discovery signal; uncultured candidate
30	Intervention study	Broilers / LPS challenge	A. muciniphila preparation + microbiome/transcriptome	Multi-omics response to intervention	3	In vivo pathway engagement; causal perturbation incomplete
31	Comparative intervention	Broilers / NSP challenge	Matched live probiotic vs postbiotic + multi-omics	Live form more strongly reduced inflammatory pathways	3	In vivo pathway engagement with a matched live-versus-postbiotic comparison suggesting viability-dependent effects; pathway-specific necessity or sufficiency was not established, so the study was not classified as Level 4 causal perturbation.
32	Engineered living therapeutic	Broilers / subclinical necrotic enteritis	Engineered *L. reuteri* nanobody vectors + metatranscriptomics/host expression/performance	Improved productivity with in situ programmed activity	5	Illustrates controlled target-species efficacy with programmed activity; full cumulative Level 5 depends on the complete preceding identity/safety/formulation package, and Level 6 field/ecological readiness remains incomplete.
33	Postbiotic intervention	Broilers / necrotic enteritis	*L. reuteri* postbiotic intervention	Growth and intestinal-health improvement	N/A	Target-animal efficacy evidence for a non-live postbiotic intervention; retained as complementary postbiotic evidence and not mapped to the live-candidate evidence ladder.
34	Host-derived intervention	Goat kids / diarrhea	*C. beijerinckii* R8 + microbiota/barrier/immune outcomes	Reduced diarrhea-related outcomes	3	In vivo target-animal response; direct perturbation/exposure tracking incomplete

35	Host-derived intervention	Early-weaned lambs	L. johnsonii M5 + antioxidant/immune/intestinal outcomes	Improved health and diarrhea outcomes	3	In vivo target-animal response; causal perturbation/field replication incomplete
36	Boundary-sector systematic review	Aquaculture	Systematic review	Probiotic/prebiotic/synbiotic health and water-quality evidence	N/A	Boundary evidence only
37	Boundary-sector scoping review	Mariculture	Scoping review	Research trends, gaps and ecological considerations	N/A	Boundary evidence only
38	Formulation review	General/strict anaerobes	Microcomposite delivery review	Delivery constraints for extremely oxygen-sensitive NGPs	N/A	Manufacturing/formulation evidence
39	Safety characterization	Industrial probiotic strains	Phenotypic + genomic AMR characterization	Illustrates genotype/phenotype safety assessment	1	Identity/safety evidence; not a target-species efficacy study
40	Regulatory guidance	EU food chain	EFSA guidance	Microorganism characterization for risk assessment	N/A	Authoritative regulatory/safety guidance
41	Regulatory framework	EU	EFSA QPS resource	QPS pre-assessment approach	N/A	Authoritative regulatory context
42	Regulatory guidance	United States / human LBP	FDA guidance	CMC expectations for human live biotherapeutics	N/A	Transferable regulatory concept; not veterinary guidance
43	Regulatory guidance	United States / animal feed	FDA CVM compliance guide	Direct-fed microbial product context	N/A	Veterinary regulatory context
44	Regulatory guidance	United States / animal food	FDA web guidance	Animal food labeling and disease-claim context	N/A	Veterinary regulatory context
45	Regulation	European Union / animal nutrition	EU Regulation 1831/2003	Feed additive regulatory framework	N/A	Authoritative regulatory context

Reference list corresponding to Table S1

1. Hill, C.; Guarner, F.; Reid, G.; Gibson, G.R.; Merenstein, D.J.; Pot, B.; Morelli, L.; Canani, R.B.; Flint, H.J.; Salminen, S.; et al. The International Scientific Association for Probiotics and Prebiotics consensus statement on the scope and appropriate use of the term probiotic. Nat. Rev. Gastroenterol. Hepatol. 2014, 11, 506–514. https://doi.org/10.1038/nrgastro.2014.66.
2. O'Toole, P.W.; Marchesi, J.R.; Hill, C. Next-generation probiotics: The spectrum from probiotics to live biotherapeutics. Nat. Microbiol. 2017, 2, 17057. https://doi.org/10.1038/nmicrobiol.2017.57.
3. Kern, L.; Tofield, A.; Frame, J.; Elinav, E. Next-generation probiotics: An outlook into current applications and future developments. Nat. Rev. Microbiol. 2026, 24, 539–558. https://doi.org/10.1038/s41579-026-01311-0.
4. Mârza, S.M.; Munteanu, C.; Papuc, I.; Radu, L.; Purdoiu, R.C. The role of probiotics in enhancing animal health: Mechanisms, benefits, and applications in livestock and companion animals. Animals 2025, 15, 2986. https://doi.org/10.3390/ani15202986.
5. Wang, Z.; Wang, W.; Zhang, P.; Luo, J.; Zhang, Y.; Yang, Q. From conventional probiotics to precision nutrition: Host-specific probiotics in canine and feline functional foods. Front. Vet. Sci. 2026, 13, 1881134. https://doi.org/10.3389/fvets.2026.1881134.
6. Al-Surrayai, T.; Shreaz, S.; Al-Khalaifah, H. Next-generation probiotics in poultry: From conventional strains to engineered and synbiotic approaches. Microorganisms 2026, 14, 1680. https://doi.org/10.3390/microorganisms14081680.
7. Salminen, S.; Collado, M.C.; Endo, A.; Hill, C.; Lebeer, S.; Quigley, E.M.M.; Sanders, M.E.; Shamir, R.; Swann, J.R.; Szajewska, H.; et al. The International Scientific Association of Probiotics and Prebiotics (ISAPP) consensus statement on the definition and scope of postbiotics. Nat. Rev. Gastroenterol. Hepatol. 2021, 18, 649–667. https://doi.org/10.1038/s41575-021-00440-6.
8. Nyholm, L.; Koziol, A.; Marcos, S.; Botnen, A.B.; Aizpurua, O.; Gopalakrishnan, S.; Limborg, M.T.; Gilbert, M.T.P.; Alberdi, A. Holo-omics: Integrated host-microbiota multi-omics for basic and applied biological research. iScience 2020, 23, 101414. https://doi.org/10.1016/j.isci.2020.101414.
9. Rogers, A.B.; Kale, V.; Baldi, G.; Alberdi, A.; Gilbert, M.T.P.; Gupta, D.; Limborg, M.T.; Li, S.; Payne, T.; Petersen, B.; et al. HoloFood Data Portal: Holo-omic datasets for analysing host-microbiota interactions in animal production. Database 2025, 2025, baae112. https://doi.org/10.1093/database/baae112.
10. Chen, C.-C.; Pilla, R.; Toresson, L.; Sung, C.-H.; Blake, A.B.; Correa Lopes, B.; Turck, J.; Jergens, A.E.; Summers, S.C.; Unterer, S.; et al. Microbial gene profiling and targeted metabolomics in fecal samples of dogs with chronic enteropathy with or without increased dysbiosis index. J. Vet. Intern. Med. 2025, 39, e70199. https://doi.org/10.1111/jvim.70199.
11. Feng, T.; Wu, Y.; Xu, Y.; Chen, W.-H. A comprehensive ruminant microbial catalog (CRMC) reveals convergent selection for key vitamin-synthesizing pathways and genes across ruminants and human. GigaScience 2026, 15, giag016. https://doi.org/10.1093/gigascience/giag016.
12. Chuang, S.-T.; Chen, C.-T.; Hsieh, J.-C.; Li, K.-Y.; Ho, S.-T.; Chen, M.-J. Development of next-generation probiotics by investigating the interrelationships between gastrointestinal microbiota and diarrhea in preruminant Holstein calves. Animals 2022, 12, 695. https://doi.org/10.3390/ani12060695.
13. Das, A.; Behera, R.N.; Kapoor, A.; Ambatipudi, K. The potential of meta-proteomics and artificial intelligence to establish the next generation of probiotics for personalized healthcare. J. Agric. Food Chem. 2023, 71, 17528–17542. https://doi.org/10.1021/acs.jafc.3c03834.
14. Correa Lopes, B.; Chen, C.-C.; Sung, C.-H.; Ishii, P.E.; Medina, L.F.D.C.; Gaschen, F.P.; Suchodolski, J.S.; Pilla, R. Correlation between Peptacetobacter hiranonis, the baiCD gene, and secondary bile acids in dogs. Animals 2024, 14, 216. https://doi.org/10.3390/ani14020216.
15. Huang, H.-W.; Kuo, T.-C.; Lee, Y.-J.; Chen, M.-J. Multi-omics analysis reveals the efficacy of two probiotic strains in managing feline chronic kidney disease through gut microbiome and host metabolome. Front. Vet. Sci. 2025, 12, 1590388. https://doi.org/10.3389/fvets.2025.1590388.
16. Glass, E.M.; Kolling, G.L.; Papin, J.A. Genome-scale metabolic modelling identifies vaginal microbiome members as potential probiotics. Nat. Microbiol. 2026, 11, 2034–2046. https://doi.org/10.1038/s41564-026-02380-w.
17. Song, H.; Mun, S.-H.; Han, D.-W.; Kang, J.-H.; An, J.-U.; Hwang, C.-Y.; Cho, S. Probiotics ameliorate atopic dermatitis by modulating the dysbiosis of the gut microbiota in dogs. BMC Microbiol. 2025, 25, 228. https://doi.org/10.1186/s12866-025-03924-6.
18. Huang, H.-W.; Yeh, T.-C.; Hsieh, J.-C.; Tsai, C.-W.; Lee, Y.-J.; Chen, M.-J. Evaluating the adjuvant therapeutic effects of probiotic strains Lactococcus cremoris and Lacticaseibacillus paracasei on canine atopic dermatitis and their impact on the gut and skin microbiome. Animals 2025, 15, 3098. https://doi.org/10.3390/ani15213098.
19. Pacheco, R.C.F.; Lins, L.F.; de Brito, L.P.; de Andrade Calaça, P.R.; Porto, A.L.F.; Cavalcanti, M.T.H. Probiotics as an adjunct in the treatment of canine atopic dermatitis: A systematic review and meta-analysis of in vivo studies in dogs. J. Vet. Med. Sci. 2025, 87, 1068–1077. https://doi.org/10.1292/jvms.24-0471.
20. Nicolas, C.S.; Lloret, F.; Carton, T.; Beuvin, L.; Rème, C.A. Beneficial effects of a prebiotic-postbiotic supplement on digestive health and fecal microbiota in dogs and cats. Front. Vet. Sci. 2026, 13, 1797178. https://doi.org/10.3389/fvets.2026.1797178.
21. Bonel-Ayuso, D.P.; Pineda-Pampliega, J.; Martinez-Alesón García, P.; Fernandez-Muela, M.; de la Fuente, J.; Garcia Fernandez, P.M.; Isabel Redondo, B. Effects of postbiotic administration on canine health: A systematic review and meta-analysis. Microorganisms 2025, 13, 1572. https://doi.org/10.3390/microorganisms13071572.
22. Ericsson, A.C. A comprehensive review of the equine gut microbiome in health and disease. Vet. Sci. 2026, 13, 659. https://doi.org/10.3390/vetsci13070659.
23. Chamtim, P.; Raethong, N.; Thananusak, R. Functional profiling of antimicrobial resistance in rabbit gut microbiome. Acta Microbiol. Hell. 2025, 70, 21. https://doi.org/10.3390/amh70020021.
24. Chuang, S.-T.; Li, K.-Y.; Tu, P.-W.; Ho, S.-T.; Hsu, C.-C.; Hsieh, J.-C.; Chen, M.-J. Investigating the reciprocal interrelationships among the ruminal microbiota, metabolome, and mastitis in early lactating Holstein dairy cows. Animals 2021, 11, 3108. https://doi.org/10.3390/ani11113108.
25. Hsieh, J.-C.; Chuang, S.-T.; Hsu, Y.-T.; Ho, S.-T.; Li, K.-Y.; Chou, S.-H.; Chen, M.-J. In vitro ruminal fermentation and cow-to-mouse fecal transplantations verify the inter-relationship of microbiome and metabolome biomarkers: Potential to promote health in dairy cows. Front. Vet. Sci. 2023, 10, 1228086. https://doi.org/10.3389/fvets.2023.1228086.
26. Hou, G.; Deng, H.; Zhou, L.; Liu, Y.; Li, W.; Li, W.; Wang, Q. Lactobacillus rhamnosus GG alleviates post-weaning stress-induced intestinal barrier damage and inflammation by promoting intestinal health and modulating the gut microbiota in piglets. Microorganisms 2026, 14, 410. https://doi.org/10.3390/microorganisms14020410.
27. Wang, Z.; Lin, Z.; Lin, B.; Peng, S.; Xu, Y.; Wang, X.; Wu, H.; Xie, B.; Chen, B.; Zhao, M.; et al. Composite probiotic fermented feed enhances growth performance and intestinal health in weaned piglets by modulating the gut microbiome and metabolome. Animals 2026, 16, 972. https://doi.org/10.3390/ani16060972.

28. Yeom, S.-J.; Yang, J.; Yoon, K.-N.; Kim, S.-S.; Cho, M.-J.; Park, J.-H.; Song, B.-S.; Park, J.H.; Jin, Y.B.; Kim, Y.-M.; et al. Safety and next-generation probiotic potential of Mitsuokella multacida AGMB01124 newly isolated from healthy swine feces. Probiotics Antimicrob. Proteins 2026, 18, 7583–7604. https://doi.org/10.1007/s12602-026-10925-y.

29. Marcos, S.; Odriozola, I.; Aizpurua, O.; Eisenhofer, R.; Mak, S.S.T.; Martin-Bideguren, G.; Kale, V.; Baldi, G.; Richardson, L.J.; Finn, R.D.; et al. Functional gut microbiota dynamics of generalist and specialist bacteria in association with chicken growth. ISME Commun. 2026, 6, ycag091. https://doi.org/10.1093/ismeco/ycag091.

30. Zhu, R.; Tang, J.; Jing, Y.; Ou, L.; Zou, Z.; Luo, C.; Feng, X. Multi-omics evaluation of Akkermansia muciniphila intervention on LPS-challenged yellow-feathered broilers. Anim. Nutriomics 2026, 3, e1. https://doi.org/10.1017/anr.2025.10019.

31. Jansseune, S.C.G.; Hendriks, W.H.; Blanc, F.; Estellé, J.; Bruneau, N.; Coville, J.-L.; Pinard-van der Laan, M.-H. A lactobacilli-based probiotic but not its postbiotic reduces intestinal inflammatory pathways expression in broilers fed a non-starch polysaccharide rich challenge diet. Poult. Sci. 2026, 105, 106159. https://doi.org/10.1016/j.psj.2025.106159.

32. Hall, A.N.; Manuja, S.; Payling, L.M.; Romero, L.F.; Hoerr, F.J.; Shields, J.; Hofacre, C.; Susanti, D.; Gangaiah, D.; Plata, G.; et al. Lactobacillus-vectored nanobodies improve broiler productivity under sub-clinical necrotic enteritis with associated microbiome and transcriptome changes. npj Biofilms Microbiomes 2026, 12, 52. https://doi.org/10.1038/s41522-026-00916-w.

33. Wu, M.; Zha, Y.; Xiong, Y.; Zhang, Y.; Zhang, Y.; Liu, J.; Yan, J.; Li, P.; Li, C.; Guo, S. Lactobacillus reuteri postbiotics improved growth performance and intestinal health of broilers with necrotic enteritis. BMC Vet. Res. 2026, 22, 32. https://doi.org/10.1186/s12917-025-05178-w.

34. Fan, D.; Fu, Y.; Zhang, J.; Bi, Y.; Ma, T.; Diao, Q.; Zhang, N. Sheep-derived butyrate-producing Clostridium beijerinckii R8 alleviates diarrhea by shaping the gut microbiota of goat kids. Anim. Nutr. 2024, 19, 13–24. https://doi.org/10.1016/j.aninu.2024.06.004.

35. Wang, Z.; Zhao, Y.; Fan, D.; Zhang, J.; Diao, Q.; Cui, K. Sheep-derived Lactobacillus johnsonii M5 enhances immunity and antioxidant capacity, alleviates diarrhea, and improves intestinal health in early-weaned lambs. Microorganisms 2025, 13, 404. https://doi.org/10.3390/microorganisms13020404.

36. Wen, Y.; Wang, M.; Wang, H.; Liu, S.; Xing, R.; Zhang, H.; Chen, L.; Li, R.; Yu, Z. Promoting aquatic animal health and water quality: A systematic review on probiotics, prebiotics and synbiotics in aquaculture. Fishes 2026, 11, 174. https://doi.org/10.3390/fishes11030174.

37. Alperstein, L.; Nappi, J.; Egan, S. Mariculture probiotics: A scoping review of research trends, knowledge gaps and opportunities. Mar. Biotechnol. 2026, 28, 152. https://doi.org/10.1007/s10126-026-10701-7.

38. Ta, L.P.; Corrigan, S.; Abeysekera, H.; Horniblow, R.D. Extremely oxygen-sensitive next-generation probiotics: Can current microcomposite formulations ensure effective colonic delivery? Microsyst. Nanoeng. 2026, 12, 37. https://doi.org/10.1038/s41378-025-01151-7.

39. Kerek, Á.; Palkovicsné Pézsa, N.; Kaszab, E.; Jerzsele, Á.; Farkas, O. Phenotypic and genotypic characterization of probiotic strains in the context of antimicrobial resistance. Front. Vet. Sci. 2025, 12, 1684650. https://doi.org/10.3389/fvets.2025.1684650.

40. EFSA Scientific Committee; Bennekou, S.H.; Allende, A.; Bearth, A.; Casacuberta, J.; Castle, L.; Coja, T.; Crépet, A.; Halldorsson, T.I.; Hoogenboom, R.; et al. Guidance on the characterisation of microorganisms in support of the risk assessment of products used in the food chain. EFSA J. 2025, 23, e9705. https://doi.org/10.2903/j.efsa.2025.9705.

41. European Food Safety Authority. Qualified presumption of safety (QPS). 2026. Available online: https://www.efsa.europa.eu/en/topics/topic/qualified-presumption-safety-qps (accessed on 6 September 2026).

42. U.S. Food and Drug Administration. Early Clinical Trials with Live Biotherapeutic Products: Chemistry, Manufacturing, and Control Information; Guidance for Industry; FDA: Silver Spring, MD, USA, 2016.

43. U.S. Food and Drug Administration. CPG Sec. 689.100 Direct-Fed Microbial Products; Center for Veterinary Medicine: Silver Spring, MD, USA, 1995.

44. U.S. Food and Drug Administration. Animal food labeling and pet food claims. 2026. Available online: https://www.fda.gov/animal-veterinary/animal-foods-feeds/animal-food-labeling-and-pet-food-claims (accessed on 6 September 2026).

45. European Parliament and Council. Regulation (EC) No. 1831/2003 of 22 September 2003 on additives for use in animal nutrition. Off. J. Eur. Union 2003, L268, 29–43.
